# Early Vascular Aging in Hypertension

**DOI:** 10.3389/fcvm.2020.00006

**Published:** 2020-02-04

**Authors:** Peter M. Nilsson

**Affiliations:** Skane University Hospital, Lund University, Malmö, Sweden

**Keywords:** aging, artery, glucose, hypertension, inflammation, oxidative stress

## Abstract

With increasing age, the cardiovascular risk increases, as does frailty, with negative health consequences such as coronary disease, stroke, and vascular dementia. However, this aging process seems to take a more rapid course in some individuals, as reflected in the Early Vascular Aging (EVA) syndrome that over the recent 10 years has attracted increased attention. The core of the EVA syndrome is arterial stiffness in the media layer of large elastic arteries, a process that can be measured by pulse wave velocity, for example, along the aorta. Hypertension is a well-known cardiovascular risk factor in its own right, but also linked to the EVA process. However, several studies have shown that non-hemodynamic factors also contribute to arterial stiffness and EVA, such as impaired glucose metabolism, chronic inflammation, and oxidative stress. New perspectives have been introduced for linking early life programming affecting new-born babies and birth weight, with a later risk of hypertension, arterial stiffness and EVA. New drugs are being developed to treat EVA when lifestyle intervention and conventional risk factor controlling drugs are not enough. Finally, the opposite phenotype of EVA is Healthy Vascular Aging (HVA) or even Super Normal Vascular Aging (SUPERNOVA). If protective mechanisms can be found and mapped in these fortunate subjects with a slower than expected aging process, there could exist a potential to find new drug targets for preventive therapy.

Patients with essential hypertension have an increased risk of cardiovascular disease (CVD), not only because of the hemodynamic burden inflicted by elevated blood pressure, but also due to the fact that hypertension often clusters with a number of cardiovascular risk factors in the same individual as described by the Lancet Commission on Hypertension 2016 (1). This was previously often referred to as the Metabolic syndrome, based on its most recent definition in 2009 with its components of abdominal obesity, dyslipidaemia, hyperglycemia, and elevated blood pressure (2). As the concept of the Metabolic syndrome has been subject to criticism ever since 2005, mostly because of the fact that the syndrome is not more that its components for risk prediction (3), there is a need to find new concepts.

The so called *Early Vascular Aging* (EVA) syndrome was first described in 2008 (4, 5) and this has been followed by a variety of studies that explored cardiovascular aging as a fruitful concept to look for new mechanisms and treatment targets. The core component of EVA is supposed to be arterial stiffness as measured by elevated carotid-femoral pulse wave velocity (c-f PWV) along the aorta, which can now be measured directly with some modern technical devices (6). Aortic distensibility can also be determined by use of ultrasound and magnetic resonance imaging (MRI). Other indirect methods try to provide an estimate of PWV, but inherent technical shortcomings may preclude researchers from making a correct assessment. In a recent study in patients undergoing coronary angiography, who thereby had their central hemodynamics measured at the same time, it was shown that most devices providing a direct measurement of central hemodynamic and aortic c-f PWV are reliable, but not all devices provided indirect measurements; for example, using age and systolic blood pressure in their algorithm for indirect estimation of aortic PWV (7).

## Determinants of Arterial Stiffness

Population-based studies have shown that close correlations exist between the level of blood pressure and the degree of arterial stiffness (aortic PWV); the higher the brachial or central blood pressure, the higher the aortic PWV (Table 1). As hemodynamic factors play an important role for the morphology and functionality of the arterial wall, there is no doubt that hypertension is a crucial factor for determining the level of arterial stiffness c-f PWV) (8). However, some studies have also documented that a number of non-hemodynamic factors, linked to glucose metabolism and chronic inflammation, are also of importance for PWV (9, 10). Prospective studies have indicated that not only can increased blood pressure predict future arterial stiffness (PWV), but that arterial stiffness can also predict incident hypertension (11, 12), as well as incident type 2 diabetes (13). This shows the close association between these entities. In fact, genetic studies have documented that a genetic risk score for hyperglycemia, as a phenotypic trait in the normal, non-diabetic, elderly population, is independently associated with arterial stiffness (c-f PWV) (14). If a true causal mechanism exists, this could imply that more focused treatment directed toward this mechanism could also alleviate arterial stiffness beyond the effect of blood pressure lowering *per se*.

**Table 1:** The links between hypertension and Early Vascular Aging (EVA) with arterial stiffness as core feature.

• Hypertension can predict arterial stiffness, but arterial stiffness can also predict hypertension.
• Alterations of the arterial wall promoting stiffness are also associated with remodeling and impaired microcirculation thereby increasing total peripheral resistance and blood pressure
• Early life may influence both vascular dysfunction (arterial stiffness) and blood pressure elevation—a common antecedent
• Treatment of hypertension will lower the degree of arterial stiffness, and new antidiabetic drugs with favorable effects on the arterial wall can lower both office as well as ambulatory blood pressure

## How to Define EVA?

The definition of EVA has recently been discussed (15), but no established definition is yet available. However, as increased c-f PWV is the core feature of EVA one can try to define EVA as the upper 10, 20, or 25% of the c-f PWV distribution in relation to a background population. For example, a reference population in Europe with PWV exists for comparison (16). Several studies and meta-analyses have documented that PWV is predictive of fatal and non-fatal CVD events, but also of total mortality (17–19). It seems that the predictive power of PWV is more pronounced in middle-aged subjects, compared to the elderly, where selective survival bias may influence the observational findings (18). Differential aging in general, and of the vasculature in particular, may be more visible in middle-aged subjects and this is linked to risk. European guidelines therefore mention the usefulness of determination of arterial stiffness (PWV), although with a lower level of evidence than, for example, the measurement of blood pressure—an established risk factor with intervention studies showing clear benefits (20). As impaired glucose metabolism is closely associated with vascular aging when measured by PWV (9, 10) it makes sense to offer the measurement of fasting glucose or even an oral glucose tolerance test (OGTT, 75 g glucose) to risk subjects, for example, following a myocardial infarction. Even if chronic inflammation seems to be of importance for EVA there is no consensus today whether inflammatory biomarkers should be measured in the clinic or not.

## Mechanisms of Importance to Modify Arterial Stiffness

Several studies have documented the association of chronic inflammation with increased arterial stiffness and PWV; for example, rheumatoid arthritis and inflammatory bowel disease, i.e., ulcerous colitis and morbus Crohn (21). It is suggested that chronic inflammation and increased oxidative stress will have a negative impact the proteins and structure of the arterial wall, but also on impaired vasodilation. A recent review has documented the importance of vascular smooth muscle cell (VSMC) changes in relation to arterial stiffness (22). These authors state that the first components that contribute to arterial stiffening are extracellular matrix (ECM) proteins that support the mechanical load, while the second important components are VSMCs, which not only regulate actomyosin interactions for contraction but mediate also so-called mechanotransduction in cell-ECM homeostasis. It seems that VSMC plasticity and signaling in both conductance and resistance arteries are highly relevant to the physiology of normal and EVA (22). This process also involves the architecture of cytoskeletal proteins and focal adhesion, the large/small arteries cross-talk that gives rise to target organ damage, and inflammatory pathways leading to calcification or atherosclerosis (22).

## Factors in Early Life Influencing Arterial Stiffness and EVA

A new aspect of EVA is the hypothesis that early life factors such as fetal growth, birth weight adjusted for gestational age, prematurity, and post-natal growth patterns, could influence both arterial stiffness and blood pressure regulation, as measured by PWV (23) or Augmentation index (Aix), another but more complex marker of stiffness and central hemodynamics, as well as total peripheral resistance (24). In one recent study from Austria these early life factors were found to be predictive of EVA in adolescents (mean age 16 years) (25). There is an ongoing debate trying to clarify whether genetic factors form the basis of the link between parental hypertension and the same trait's presence in their offspring—when low birth weight is just a marker of the trait (26)—or whether environmental factors play the most important role, i.e., maternal diet (27), calorie intake, and lifestyle (smoking, alcohol). Probably genetic factors form a background structure, whereas environmental factors can play a modifying role (epigenetics) for the phenotypic outcome.

## Vascular Aging and the Brain

Hypertension is a well-documented risk factor for stroke and other cerebrovascular disease manifestations such as microangiopathy and white matter lesions (WML), often affecting the elderly. Also, arterial stiffness can contribute to these pathologies in different ways (28, 29). One result is the impaired cognition and increased risk of dementia in these patients. Thus, hypertension and EVA may cause more damage in the population at large than visible from hospital statistics of stroke alone. If impaired cognition and dementia occurs several years earlier than in people without these risk conditions, this will substantially impact actual daily living (ADL) capacities and independence of care in aging populations.

## Treatment of EVA and Hypertension

The treatment of EVA and hypertension is based on an improved healthy lifestyle and treatment of conventional risk factors, based on current best evidence as shown in both European (20) and US guidelines (30). Several observational studies have indicated that antihypertensive treatment may reduce arterial stiffness (PWV) beyond the blood pressure reduction itself, when blockers of the renin-angiotensin-aldosterone system (RAS) in particular seem to be of great value (31). Recently, in the SPRINT study an estimated PWV (ePWV) was shown to be lowered by relatively more than the intensive blood pressure control implicated in the intervention arm (32). These results suggest that, in this trial, ePWV predicted outcomes independent of the Framingham Risk Score (where blood pressure is included), indicating an incremental role of markers of aortic stiffness on cardiovascular risk. The authors concluded that better survival of individuals whose ePWV responded to antihypertensive treatment independently of systolic blood pressure reduction suggests a role of markers of aortic stiffness as effective treatment targets in individuals with hypertension (32).

The ultimate proof of the usefulness of PWV for risk stratification and as a target for therapy will come with the SPARTE study in France where risk individuals have been randomized to either a treatment strategy aimed to lower PWV or to conventional treatment aiming for multiple risk factor intervention and control based on guidelines (33). SPARTE has been underway for a few years and the results should be reported during 2020.

Interestingly enough, it has been shown that newer anti-diabetes drugs such as the SGLT-2 inhibitor empagliflozin may lower both office and ambulatory blood pressure (34), and in addition show beneficial effects on Aix—a marker of aortic dysfunction—in patients with type 2 diabetes (35).

## Look for Protection, Not Only Risk!

A very novel aspect of EVA is to turn it around and look for factors that protect from EVA and are associated with Healthy Vascular Aging (HVA) (36–38), or even Super Normal Vascular Aging (SUPERNOVA) (39). If such protection from vascular aging could be better defined and understood, based on advanced phenotyping using genetics and omics, there is a potential to find new drug targets of protection. Also, other models of protection exist, but are poorly understood; for example the astonishing lack of major complications in a few patients with type 1 diabetes with more than 40–50 years of diabetes (40–42). It would be interesting to further examine vascular function and the escape from hemodynamic aging in these fortunate subjects (43). Another example includes obese subjects who were not hospitalized for decades in mid-life in spite of risk factors and drug treatment (44, 45). Some of them seem to be “fat and fit” as a way to cope with obesity and its risks. Even if obesity is a strong risk factor for the development of type 2 diabetes, many of these subjects with “Metabolically Healthy Obesity” (HMO) escape diabetes. Even if they are not thought to be protected from complications in the long run, such HMO subjects may benefit from a postponement of complications for a substantial time.

## Conclusions

In conclusion, the EVA concept has enriched translational research activities to find new mechanisms of CVD risk (46) and also inspired researchers to find new treatment targets to further lower the CVD risk beyond what can be achieved by conventional risk factor control. Screening of EVA has been tried based on measurements at pharmacies offered to the public (47) and seems to be feasible. The most recent development is focused on the opposite of EVA, namely, Healthy Vascular Aging (HVA) or even Super Normal Vascular Aging (SUPERNOVA) in order to understand vascular protection and find new treatment targets.

## Author Contributions

The author confirms being the sole contributor of this work and has approved it for publication.

### Conflict of Interest

The author declares that the research was conducted in the absence of any commercial or financial relationships that could be construed as a potential conflict of interest.
